# Safety in Healthcare: From the Flight Deck to the Operating Room

**DOI:** 10.4274/TJAR.2023.231264

**Published:** 2023-08-18

**Authors:** Régis Fuzier, Philippe Izard, Eric Petiot, François Jaulin

**Affiliations:** 1 Department of Anesthesiology, Claudius Regaud Institute, Cancer University Institute-Oncopole, Toulouse, France; 2 Captain and Crew Resource Management Trainer, Air France Airlines, Paris, France; 3 French Association on Human Factors in Healthcare, Paris, France

**Keywords:** Check-list, cross-check, flow, human factors, readback

## Abstract

The recent health crisis has increased the workload and the stress levels of healthcare professionals around the world. Such stressful working environments are conducive to an increased incidence of medical errors. Implementing education and training specifically focused on human and organizational factors can promote teamwork and decrease the risk of error. Such techniques have been extensively deployed, most notably in commercial aviation. Numerous tools have been developed to reduce the risk of error associated with routine tasks, forgetting a task and handling alarm situations during commercial flights. Many of these tools can be transferred to the healthcare sector. After a brief recap about the importance of the working environment, this narrative review aims to highlight several specific tools used in commercial aviation that can be readily transferred to the operating theatre.

**Main Points**

• To improve quality and safety in healthcare environment, it is important to integrate the work on human factors into everyday practice.

• Based on the aeronautics’ experience, it therefore seems conceivable to adapt some of these procedures to the healthcare sector.

• The development of non-technical skills (various tools, communication techniques, and teamwork simulation) training courses should be encouraged at the very early training stages of healthcare professionals.

• These tools must be used routinely on a daily basis, in order to be even more effective in stressful situations.

• This review provides useful tools that can be easily and quickly implemented in our healthcare institutes.

## Introduction

At a time when the world is faced with the Coronavirus disease-2019 pandemic, healthcare professionals are confronted with work overloads in the context of increased stress levels. These specific work conditions increase the risk of medical errors. In fact, medical errors represent the 3^rd^ leading cause of death in the United States. The vast majority of medical errors do not result from an individual action, but rather from the collective actions, of teams, systems and/or procedures.^1^ A lack of awareness of the importance of Human Factors (or more accurately Organizational and Human Factors) as the primary cause of healthcare complications is no longer a matter of debate.^2,3^ It is therefore becoming increasingly important to develop non-technical skills to improve safety in the field. Lessons drawn from a range of industries (aeronautics, nuclear power plants, car industry, etc.) can be applied to the medical field, and particularly to surgical and anaesthetic activities.^4^ The aviation safety model is the one most often cited as example. Lessons learned from aviation may help reduce operating theatre errors.^5^ These practices would need to be adapted by healthcare professionals and tailored to their specific practices.^3^ The reason model defines several different types of errors.^6^ Specialists are particularly susceptible to routine errors, which occur whilst performing routine tasks or simply forgetting to perform a task. The management of alarms also warrants a mention. The objective of this article is to present tools used in commercial aviation that can be readily transferred to the operating room, but also more generally to the healthcare institutions themselves.

### The Working Environment

The working environment is a significant independent factor that increases the risk of errors.^7^ Numerous components of the working environment can indeed disrupt the ability to concentrate whilst performing a given task, increase the risk of becoming distracted and thereby increase the likelihood of an error occurring. Disturbing any one of the five senses of an operator may affect a healthcare worker’s performance in the operating theatre.

In terms of the auditory system, surgery generates many different types of sounds (electric scalpel, electric syringe, suction, ventilation, alarms, telephone, discussions between operators, etc.). Background music is also often played during the operation. The type of music played may induce sleepiness, apathy or conversely excitement. Excessively loud music can interfere with communication, resulting in a word or a phrase being misunderstood or not heard at all. Conversations that are not related to the actual procedure (external phone calls, non-medical discussions, etc.) may also increase the risk of distraction. Smartphone use and the management of personal or professional messages have recently been identified as independent risk factors of reduced vigilance.^8,9^ These risks are compounded by lack of sleep, fatigue, taking narcotic drugs or other specific drugs.^10,11,12^ Factors such as hunger and particularly dehydration, further increase these risks (the recommendation in the aviation industry is to drink 1 litre of water every 4 flight hours).

The commercial aviation industry, limits communications below 10,000 feet - approximately 3,000 m (in non-emergency situations). This is commonly known as the “sterile cockpit rule”.^13^ The choice of words is both specific and conventional. In terms of the visual system, several sources of light may be used in an operating theatre (surgical lamp, ceiling light, desk lamp, lights emitted by computer screens or medical devices, etc.) and may impact performance. Healthcare workers are also exposed to night shifts and sleep deprivation.

These same issues have been investigated in aeronautics. The time allocation of pilots’ working hours takes into account biological rhythms and disruptions caused by some types of flights, in particular long-haul flights, and their impact on sleep patterns.

In Europe, work hours in the medical field are defined by European regulations.^14^ However in practice, this regulation is not integrated into healthcare organization, with many healthcare workers doing shifts of longer than 12 hours straight, for more than 48 hours a week.^15^ The work hours of professional pilots, regulated by the International Civil Aviation Organization, are limited to a fixed number of hours per rolling week: regardless of the actual date, their time on duty never exceeds 60 hours over the previous 7 days.^16^ Both in-flight and land-based hours are taken into account and there is little margin for exceeding these hours. Some flights may even be diverted from their final destination to comply with these working hours regulation (on 22 February 2015, flight AF007 from Paris to New-York, was for instance, diverted via Manchester).

### Routine Tasks

There are many routine tasks that need to be performed during a flight (entering data into the on-board computer, changing radio frequency, setting heading and altitude, adjusting the altimeter setting, performing weight and balance calculations, etc.). Many of these tasks, which are repeated several times a day, can have devastating consequences if carried out incorrectly. Several procedures have been implemented to limit the risk of such errors. First and foremost, certain instruments are fitted with “mistake proofing” functions. This ensures that any input that does not correspond to the expected value is rejected by the on-board computer. It is also common practice to keep the last communication frequency with the controller on stand-by until a new communication has been established on the new frequency. Cross-checks are another technique which is used to mitigate the risk of error when setting heading, altitude and speed. It requires any changes to these parameters made by the Pilot on Duty (PD) to be confirmed by the Monitoring Pilot. For altimeter settings, checking the concordance of the two altimeter readings is done systematically. Most of these different measures can also be applied to healthcare.

The increasingly widespread use of electronic medical records, including in the operating theatre, may help to improve safety. This type of software should for instance incorporate the option of rejecting absurd entries (e.g., to prevent reversing the patient’s weight and height). It is likewise feasible to envisage color-coded caps on vials to eliminate mix-ups during the preparation of specific drugs. Cross-checks may also be readily deployed in the healthcare sector. When a nurse at the Cancer University Institute programs a morphine PCA pump, he/she systematically asks a colleague to confirm that the settings correspond to the prescription. The same applies to the final check of blood type matching before blood transfusions. Similarly, when a nurse assists a healthcare professional and transfers a drug from its original packaging, in addition to reading the name of the drug out aloud, the ampoule or vial label is also systematically shown to the operator. This practice has recently averted the injection of water for injection instead of saline. One last example involves the concordance of components, namely when preparing medicines before anaesthesia. The vial (or ampoule) is kept until the label stating the name and dilution of the medicine has been placed on the syringe and checked against the label of the vial. This practice ought to be generalized for all syringe and infusion bag labelling. It allows to check that the names are consistent and to identify any potential errors before administration to the patient.

### Forgetting a Task

Forgetting a task can have dramatic consequences in certain circumstances. In aeronautics, for example, there are key actions that may result in an accident, if not carried out correctly. Examples include forgetting to extend flaps to the take-off position before taxiing on the runway, forgetting to switch on the engine anti-icing devices in freezing conditions (risk of engine shutdown), forgetting to activate the approach phase during an instrument landing or forgetting to extend the landing gear on the final approach. Accidents caused by such lapses have prompted airlines to put in place procedures to limit the risk of such oversights occurring. The use of checklists is the best-known recourse. The aim of a checklist is to list only a few items, which are deemed essential at specific stages of the flight, and which if forgotten could have potentially dangerous consequences. Manufacturers and airlines have also introduced flows at key stages of the flight. These flows consist of carrying out the same actions, in a set order that remains unchanged, during different phases of the flight for each of the pilots. The repetitive and immutable nature of these flows limits the risk of forgetting an action, even in the presence of disruptive external factors. The system is further complemented with color-coded and audible alarms to help pilots recall their tasks. During maximum thrust at take-off, for instance, an alarm will sound and a red message will appear (warning) if the flaps are not in the correct position, prompting the pilots to abort take-off. The same applies during the final approach, in the event of a landing gear extension oversight (indicator light for each landing gear and audible alarm), triggering a go-around by the pilot before touch down. And finally, when pilots communicate with each other or with the air traffic controllers, the principle of collation is applied. This consists of banning answers such as “yes”, “ok” and “agreed” in response to an order. Instead, the information must be repeated, so that the instructing party can ascertain that the recipient of the information has understood the message. This collation principle applies to numerous phases of flight: take-off clearance, heading or altitude changes, clearances etc.

The risk of forgetting a task and its potentially harmful consequences also exists in the healthcare sector. In the operating room, before a general anaesthetic, forgetting to connect the suction, start monitoring, open the oxygen flowmeter, check the functionality of the peripheral venous line, connect the capnograph or adjust the hypnotic agent after induction, are just a few such examples. The frequency of task interruptions is an important component to consider as an additional risk factor which may increase the risk of oversight. These task interruptions are related to interactions between the different participants in an operating room, but also with external parties (colleagues in the operating room, telephone calls, etc.).

Most of the aeronautical tools used and presented at the beginning of this review can be applied to the healthcare sector. The World Health Organization (WHO) has made it compulsory to use a checklist, at specific times, in operating theatres since 2009:^17^ before inducing anaesthesia, before surgical incision, and at the end of the operation. To be effective, these checklists must be carried out during an adequate pause and each individual team member must feel implicated by items listed. Where a team considers it necessary, and bearing in mind the reservations mentioned above, it is possible to incorporate a number of additional specific items to these lists. A recent study concluded that the implementation of a specific check-list to standardize handover process in adult patients post-surgery was associated with a reduction in the rate of hypoxemic events in the post-anaesthesia care unit.^18^ It is also important to put in place safeguards to limit the risk of task interruptions. In some institutions, staff wear obvious badges when performing specific procedures (e.g. preparation of chemotherapy), which prompts those around them not to interrupt. It is important to train healthcare workers to refuse to be interrupted. One such practice involves raising one’s hand to say “wait” or “I am not available right now” or “stand-by” all at the same time. It is also important to be familiar with the rules which apply to interrupting tasks: Knowing when to interrupt (this is essential), knowing how to help the person get back to where they were at the time of the interruption, etc.

The use of flows may also be of interest to the medical field. A visual circuit pointing to blood pressure, oxygen saturation, capnography curve and maintaining anaesthesia ensures that the induction phase of anaesthesia is completed without any complications. A flow can also be used to double check all the connections of a patient’s ventilator circuit, from the intubation tube (or laryngeal mask) to the ventilator. Flows may also be applied to connecting an electric syringe pump.It is common practice to give numerous orders to paramedics verbally. To ensure that the information has been correctly understood, we recommend using collation. This means that when a drug is administered, the person receiving the command responds by repeating the name and dosage of the drug, and not simply by saying “ok” or “right”.

### Managing Alarms

Alarm management is a specific situation associated with a higher risk of error because it occurs in an emergency context, when stress-levels are increased. Rushing may increase the risk of precipitated decision making and may have final repercussions that are more or less disconnected from the initial objectives. In aeronautics, the first thing taught in such situations is to start by doing nothing and to breathe calmly and deeply for a few seconds, in order to re-equilibrate the sympathetic and parasympathetic nervous systems.There are two types of alarms in the cockpit: “warnings” and “cautions”. “Warning” alarms refer to urgent situations that require a certain number of actions to be taken quickly (e.g. jet engine fire). These alarms are indicated by a “red” signal and a distinctive sound. In all cases, the audible alarm should be switched off after the cause has been identified. This enables the alarm to be triggered again in the event of a new incident.

Training on a simulator allows pilots to acquire good practices. Situational awareness is integrated into performance (e.g. managing the flight path to a safe altitude before dealing with the engine fire that occurred on take-off). Each pilot has a well-defined role. Once the first actions are completed and the situation is under control, a number of actions are carried out with the help of “do-lists” which are used to complete the procedure during the secondary phase. The pilots then perform a comprehensive analysis of the situation with team decision support [Time, Facts, Options, Risks, Decision, Execution and Checking (T-FORDEC) of some airlines] taking into account T-FORDEC the implementation. Informing the controller services, the airline, the crew and the passengers is all part of the procedure. These situations are the most stressful. The triggering of a “caution” alarm represents a reduced level of urgency. The signal is visual (amber color) and a single sound is normally emitted. It indicates a situation requiring a rapid response. Generally, the PD manages the aircraft’s flight path and communications with the controllers, while the second pilot manages the alarm itself. The second pilot immediately refers to do-lists and ensures that the PD is kept in the loop. The procedure is again completed by a comprehensive analysis of the situation (such as T-FORDEC as previously described).

A similar management of alarms may be envisaged in the healthcare sector. Some healthcare procedures require a rapid response, the equivalent of a warning alarm (e.g. cardiac arrest in the operating theatre). This is when technical skills take over. Once the alert has been raised and resuscitation initiated, the use of “do-lists” may guide the clinicians’ diagnostic and therapeutic processes (e.g. by evoking some of the diagnoses applicable after cardiac arrest). The procedure could be completed with the equivalent of a “T-FORDEC” process among all the team members (stopping or continuing the surgical procedure, hospitalization in intensive care, etc.).

There are other healthcare situations which would be more akin to triggering a “caution” alarm (e.g., a power outage of the monitoring system, a drop in fluid pressure, a progressive drop in capnography, etc.). The use of a “do-list” right at the beginning of the procedure would avert the oversight of an important component. The division of tasks (such as management and monitoring of anaesthesia on the one hand and management of the alarm on the other) is perfectly feasible when reinforcements arrive. A discussion on whether or not to continue the procedure and the subsequent transfer of the patient to the appropriate department would complete the process, similarly to aviation type “warning” alarms. Improvements in cognitive ergonomics should be envisaged in healthcare, as alarms are currently not defined at all or are not very clearly defined and are therefore prone to being misinterpreted or of not being clearly understood.

### General Discussion

Many tools that focus on managing human factors have been developed to improve safety in commercial aviation. Broadly speaking, the question arises as to whether these tools can also be applied to the healthcare sector.^19^ Some teams believe that aviation is not a good model for improving safety in healthcare.^20^ Other teams, conversely, go further and stress the importance of digitizing surgical checklists on giant screens, based on current practices in aeronautics.^21^ This review attempts to illustrate the benefits of adapting safety culture practices from commercial aviation to healthcare teamwork (Table 1). One of the main research studies addressing this issue was published some ten years ago and demonstrated that using checklists prior to several key stages of surgery decreased perioperative morbidity and mortality.^17^ It resulted in the WHO making checklists mandatory in the operating theatre. Subsequent studies later validated the importance of checklists.^22^

In addition to checklists, a number of other tools from aeronautics have been tested in the healthcare sector. This has been the case for debriefings, incident analyses (feedback) and the implementation of team-based resource management [i.e., crew resource management, (CRM)].^23^ A meta-analysis specifically demonstrated the positive impact of CRM training on teamwork in the healthcare sector.^24^ Other studies have highlighted the benefits of CRM training for anaesthetic teams in terms of improving performance and reducing errors in the operating theatre.^25^ The growing interest in further developing CRM training has been confirmed in recent years. There are, however, many different approaches to CRM training in the healthcare sector.^26^

More recent publications have specifically focused on determining the impact of the normalization of deviance, the Swiss cheese model^27^ as well as the issue of threats and errors.^28^ Accident reporting procedures, such as those advocated in aeronautics by the National Transportation Safety Board, and the use of simulations have been introduced and encouraged, particularly in the field of anaesthesia.^29^

It is therefore important to focus on team training as part of this process. A one-day CRM training session can already improve non-technical skills.^1^

These practices must be complemented by procedural improvements. The importance of standardized protocols for the administration of drugs, the management of analgesia, the management of an emergency such as cardiac arrest, etc., has indeed been emphasized.^30,31,32^ Similarly, electronic systems which issue reminders (e.g. for administering antibiotics) or alerts for allergies or anomalies in the laboratory work-up may also be useful.^33,34^

## Conclusion

Given that human error is primarily a loss of human performance in the working environment, it is important to integrate the work on human factors into everyday practice. There is a real need for systems to limit the consequences of these errors. This is exactly the premise upon which CRMs, various other tools, communication techniques, and teamwork simulations have been based in the initial and ongoing training of pilots. The experience gained from aeronautics, has enabled to reduce the risk of the consequences of human errors, it therefore seems conceivable to adapt some of these procedures to the healthcare sector, and also to the initial and ongoing training of healthcare professionals. The development of non-technical skills training courses should be encouraged at the very early training stages of healthcare professionals. These new tools must be used routinely on a daily basis, in order to be even more effective in stressful situations. Ultimately, these approaches are expected to reduce the incidence and impact of medical errors. This review provides useful tools that can be easily and quickly implemented in our healthcare institutes.

## Figures and Tables

**Table 1:**
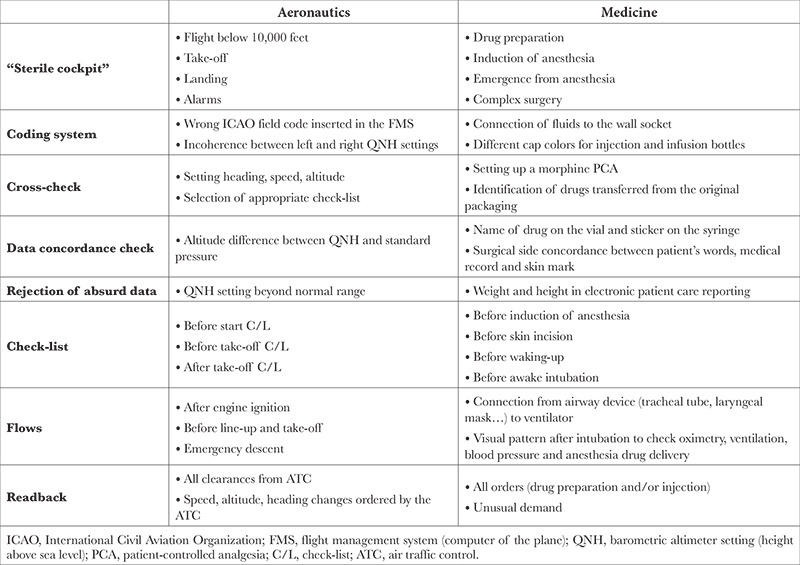
Examples of Tools Derived From Aeronautics that are Applicable to the Operating Room
